# Improved pharmacokinetics of tenofovir ester prodrugs strengthened the inhibition of HBV replication and the rebalance of hepatocellular metabolism in preclinical models

**DOI:** 10.3389/fphar.2022.932934

**Published:** 2022-08-29

**Authors:** Xiaodan Hong, Zuhuan Cai, Fang Zhou, Xiaoliang Jin, Guangji Wang, Bingchen Ouyang, Jingwei Zhang

**Affiliations:** ^1^ Key Laboratory of Drug Metabolism and Pharmacokinetics, State Key Laboratory of Natural Medicines, China Pharmaceutical University, Nanjing, Jiangsu, China; ^2^ Affiliated Hospital of Nanjing University of Chinese Medicine, Nanjing, Jiangsu, China.

**Keywords:** tenofovir, ester prodrug, anti-HBV activity, metabolomics, lipidomics, pharmacokinetics

## Abstract

Tenofovir (TFV) ester prodrugs, a class of nucleotide analogs (NAs), are the first-line clinical anti-hepatitis B virus (HBV) drugs with potent antiviral efficacy, low resistance rate and high safety. In this work, three marketed TFV ester drugs, tenofovir disoproxil fumarate (TDF), tenofovir alafenamide fumarate (TAF) and tenofovir amibufenamide fumarate (TMF), were used as probes to investigate the relationships among prodrug structures, pharmacokinetic characteristics, metabolic activations, pharmacological responses and to reveal the key factors of TFV ester prodrug design. The results indicated that TMF and TAF exhibited significantly stronger inhibition of HBV DNA replication than did TDF in HBV-positive HepG2.2.15 cells. The anti-HBV activity of TMF was slightly stronger than TAF after 9 days of treatment (EC_50_ 7.29 ± 0.71 nM vs. 12.17 ± 0.56 nM). Similar results were observed in the HBV decline period post drug administration to the HBV transgenic mouse model, although these three TFV prodrugs finally achieved the same anti-HBV effect after 42 days treatments. Furthermore, TFV ester prodrugs showed a correcting effect on disordered host hepatic biochemical metabolism, including TCA cycle, glycolysis, pentose phosphate pathway, purine/pyrimidine metabolism, amino acid metabolism, ketone body metabolism and phospholipid metabolism. The callback effects of the three TFV ester prodrugs were ranked as TMF > TAF > TDF. These advantages of TMF were believed to be attributed to its greater bioavailability in preclinical animals (SD rats, C57BL/6 mice and beagle dogs) and better target loading, especially in terms of the higher hepatic level of the pharmacologically active metabolite TFV-DP, which was tightly related to anti-HBV efficacy. Further analysis indicated that stability in intestinal fluid determined the actual amount of TFV prodrug at the absorption site, and hepatic/intestinal stability determined the maintenance amount of prodrug in circulation, both of which influenced the oral bioavailability of TFV prodrugs. In conclusion, our research revealed that improved pharmacokinetics of TFV ester prodrugs (especially intestinal stability) strengthened the inhibition of HBV replication and the rebalance of hepatocellular metabolism, which provides new insights and a basis for the design, modification and evaluation of new TFV prodrugs in the future.

## 1 Introduction

**FIGURE 1:**
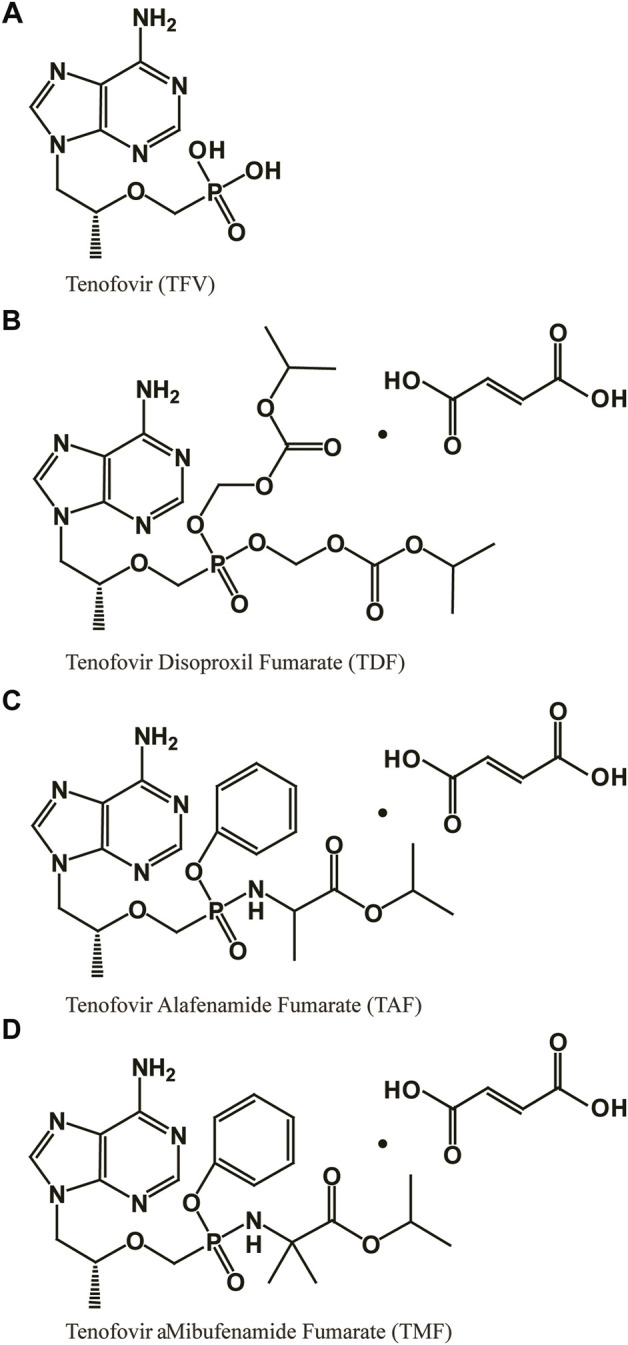
Structure of **(A)** tenofovir (TFV), **(B)** tenofovir disoproxil fumarate (TDF), **(C)** tenofovir alafenamide fumarate (TAF), and **(D)** tenofovir amibufenamide fumarate (TMF).

Hepatitis B virus (HBV) infection is a severe and worldwide public health problem that can cause chronic hepatitis and advanced-stage liver diseases, such as fibrosis, cirrhosis and even hepatocellular carcinoma (Levrero and Zucman-Rossi, 2016). According to the World Health Organization, approximately 257 million people live with chronic HBV infections and nearly 1 million deaths are caused by HBV around the globe annually (Martinez et al., 2020; Smolders et al., 2020). Nucleos(t)ide analogs (NAs) (Fung et al., 2011) and interferons (IFNs) (Kang et al., 2015) are currently approved therapies for HBV. NAs, e.g., lamivudine, adefovir, entecavir and tenofovir (TFV; Figure 1A), provide more effective suppression of HBV replication, better tolerance and greater safety than do IFNs (Lau et al., 2005), clinically benefiting HBV-infected patients.

TFV, an acyclic nucleoside phosphonate agent, exhibits potent and broad antiviral activity *in vitro* through only two-steps phosphorylation. It bypasses the first phosphorylation step due to its already having one phosphate group (De Clercq, 2003). However, the phosphonate group contributes to its poor oral bioavailability and cell permeability, which limits its efficacy *in vivo* and even causes nephrotoxicity and bone toxicity (Cundy et al., 1998). Therefore, TFV was designed as various types of ester prodrugs in recent decades to improve its antiviral activity and reduce its adverse reactions (Beadle et al., 2019; Wang et al., 2019).

The currently marketed TFV ester prodrugs include tenofovir disoproxil fumarate (TDF), tenofovir alafenamide fumarate (TAF) and tenofovir amibufenamide fumarate (TMF). TDF (Figure 1B), the first TFV prodrug approved by the FDA, degrades rapidly after absorption and delivers TFV to the system circulation (Kearney et al., 2004; Callebaut et al., 2015), contributing to higher bioavailability (Shaw et al., 1997; Naesens et al., 1998), target loading (Shaw et al., 1997) and antiviral activity (Srinivas and Fridland, 1998; Gallant and Deresinski, 2003; Delaney et al., 2006). Unfortunately, the premature hydrolysis of TDF also results in substantial untargeted TFV accumulation after long-term treatment, which is thought to be responsible for its toxicity, such as nephrotoxicity and decrease in bone mineral density (Cooper et al., 2010; McComsey et al., 2011; Compston, 2016). Hence, another phosphonoamidate prodrug of TFV was developed and approved by the FDA in 2016, namely TAF (or GS-7340, Figure 1C). Unlike the extensive degradation of TDF, TAF is sensitive to only specific enzymes in target cells, such as cathepsin A (Cat A) in PBMCs (Birkus et al., 2007; Birkus et al., 2008) and carboxylesterase 1 (CES1) in hepatocytes (Murakami et al., 2015). Its stability in nontargeted tissues contribute to its lower systemic exposure and higher target cell loading of TFV (Lee et al., 2005; Ruane et al., 2013; Markowitz et al., 2014; Callebaut et al., 2015; Ray et al., 2016; Stray et al., 2017) with improved safety (Sax et al., 2014) and efficacy (Ruane et al., 2013; Agarwal et al., 2015). TMF (Figure 1D), the third marketed TFV ester prodrug, was modified on TAF by adding one methyl group and was approved by the National Medical Products Administration (NMPA) in China for HBV infection in 2021. TMF was reported to have greater plasma stability than TDF and comparable HBV inhibition potency with TDF in a clinical trial when its dose was only 1/30 of TDF (Zhang et al., 2021). What we can learn from the development of TFV ester prodrugs from TDF to TMF will further promote the rational design of new TFV prodrugs. Therefore, in this work, TDF, TAF, and TMF were used as probes to investigate the relationships among TFV prodrug structures, pharmacokinetic characteristics, metabolic activations, and pharmacological responses and to reveal the key factors to consider in for TFV ester prodrug design.

## 2 Materials and Methods

### 2.1 Materials

TMF, TAF, TDF, and TFV (purity >98%) were purchased from MedChemExpress (New Jersey, United States ). Tenofovir diphosphate (TFV-DP) (purity >98%) was purchased from Mason Chem, Inc. (California, United States ). Lamivudine (purity >99%) was purchased from Tokyo Chemical Company (Tokyo, Japan). HPLC-grade acetonitrile and methanol were purchased from Merck (Darmstadt, Germany). Deionized water was purified by a Milli-Q system (Millipore Corporation, Billerica, MA, United States ). All other chemicals were purchased from commercial sources and were of analytical grade.

### 2.2 Cell culture

HBV-positive HepG2.2.15 cells were obtained from the China Center for Type Culture Collection in Wuhan (Hubei, China). Cells were grown in DMEM supplemented with 10% fetal bovine serum and 100 U·ml^−1^ penicillin and streptomycin (Life Technologies, Carlsbad, CA, United States ) at 37°C with 5% CO_2_. The cells in this study were used between passages 10 and 20 and were negative for mycoplasma infection. Primary rat hepatocytes were isolated from rat liver by a modified two-step collagenase perfusion method and treated and cultured as we published previously (Kang et al., 2017). In brief, rat liver was perfused *in situ* with D-hanks’ balanced salt solution and DMEM until it turned yellow and looked mushy. Then, the rat liver was taken out, mashed into a dispersed solution, filtered through a 100 μm pore size mesh nylon filter, and then centrifuged (50 g) at 4°C for 2 min. After aspirating the supernatant, the cells were re-suspended in DMEM with equal volume of percoll solution and centrifuged (50 g) at 4°C for 2 min. Next, the cells were washed and cell viability was determined. Lastly, the cells were seeded in rat tail collagen-coated 24-well cell culture plates for experiments.

### 2.3 Anti-HBV activity *in vitro*

HepG2.2.15 cells were seeded in 96-well plates at 4 × 10^4^ cells/well, and incubated with cell culture medium containing TMF/TAF/TDF at various concentrations (ranging from 0.5 nM to 20 μM). On the 3^rd^, 6^th^, and 9^th^ days post-drug incubation, the cell culture medium was harvested for HBV DNA analysis and replaced with a freshly prepared drug-containing medium. The EC_50_ was calculated with GraphPad Prism.

### 2.4 Animals

Six-week-old male HBV transgenic mice and wild-type C57BL/6 mice (18 ± 2 g) were purchased from Beijing Vitalstar Biotechnology Co., Ltd. (Beijing, China). Sprague-Dawley rats (200 ± 20 g) were purchased from Beijing Vital River Laboratory Animal Technology Co., Ltd. (Beijing, China). Beagle dogs (8.8 ± 1.0 kg) were provided by the Yadong Laboratory Animal Research Center (Nanjing, China). All the animals were housed under controlled conditions with a 12/12 h light/dark cycle and allowed free access to food and water. HBV transgenic mice were housed in Beijing Vitalstar Biotechnology Co., Ltd. (Beijing, China). Animal studies were carried out in accordance with the Institutional Animal Care and Use Committee of Beijing Vitalstar Biotechnology Co., Ltd. (Beijing, China), and the Guidelines for Animal Experimentation of China Pharmaceutical University (Nanjing, China).

### 2.5 Anti-HBV activity *in vivo*

The HBV transgenic mouse model was built by pronuclear microinjecting a linearized DNA fragment, 1.28-fold the length of the HBV genome (genotype A, GenBank ID: AF305422.1), into the fertilized egg of a C57BL/6NCrl mouse and stably transmitted HBV to the fifth generation with high titers of HBV DNA in serum (approximately 10^7^ copies/mL). These HBV transgenic mice were randomly divided into four groups with 8 mice each. Three groups were orally administered 60.7 μmol/kg TMF, TDF or TAF for 84 days, respectively, and the last group was treated with an equal volume of normal saline once daily as a positive control (drug dosage was calculated according to the clinical TDF dosage and applied to all three drugs with equivalent molar concentrations for further comparison). Another 8 wild-type C57BL/6 mice served as a negative control. Blood samples were collected every week to monitor serum HBV DNA levels. All the samples were assayed by the laboratory department of Jiangsu Province Hospital of Chinese Medicine.

### 2.6 Metabolomics and lipidomics analysis of HepG2.2.15 cells

HepG2.2.15 cells were treated with 5 μM TMF/TAF/TDF for 3, 6, and 9 days, and then cells were collected for untargeted metabolomics analysis and targeted lipidomics analysis with metabolite identification based on LC-Q/TOF-MS as we described previously (Yan et al., 2020). The compounds involved were semi-quantified through the peak area of each compound weighted by total ion chromatography. Multivariate statistical analysis, such as principal component analysis (PCA), orthogonal partial least squares-discriminant analysis (OPLS-DA) and shared and unique structure-plot (SUS-plot) analysis, were performed by SIMCA-P software version 14.1 (Umetrics AB, Umea, Sweden). Metabolite set enrichment and pathway analysis were performed by the online software MetaboAnalyst (http://www.metaboanalyst.ca/).

### 2.7 TFV activation in hepatocytes

HepG2.2.15 cells were cultured in fresh DMEM (10% FBS) at 37°C with 5% CO_2_ and seeded in 24-well cell culture plates to approximately 70%–80% confluence. Then, the cells were incubated with TMF, TAF or TDF (5 μM) for 2, 4, 8, 12, and 24 h and collected for LC-MS/MS analysis. Similar experiments were also performed in primary rat hepatocytes.

### 2.8 Pharmacokinetic profiles

Considering the features of ester prodrugs, three common animal models (including rodent and non-rodent) were used to compare the differences of the pharmacokinetic behaviors of TFV prodrugs comprehensively. The plasma pharmacokinetics of different TFV prodrugs were investigated in SD rats (iv. 10 μmol/kg; ig. 10, 30, and 90 μmol/kg; *n* = 6, male and female for half), C57BL/6 mice (iv. 14.5 μmol/kg; ig. 60.7 μmol/kg; *n* = 6, male) and beagle dogs (iv. 16.3 μmol/kg; ig. 16.3 μmol/kg; *n* = 4, male). Blood samples were collected at 0.083, 0.25, 0.5, 1, 2, 6, 12, and 24 h postdose from rats (*via* jugular vein) and dogs (*via* forelimb vein), or at 0.083, 0.25, 1, 4, 10, and 24 h postdose from mice (two time points per mouse) into EDTA-containing tubes for LC-MS/MS analysis. Pharmacokinetic parameters were calculated by a noncompartmental model using WinNonlin Pro 6.4 (Pharsight Corporation, Mountain View, CA).

To explore the biodistribution of different prodrugs, SD rats (*n* = 6, male and female for half) were orally dosed with 30 μmol/kg TMF, TAF or TDF. Tissues including heart, liver, spleen, lung, kidney, thymus, stomach and small intestines were harvested into 50% acetonitrile immediately and homogenized separately. Tissue homogenates (W/V: 0.1 g/ml) were analyzed by LC-MS/MS.

### 2.9 Prodrug stabilities throughout the absorption process

Simulated gastric fluid (SGF) and simulated intestinal fluid (SIF) were prepared according to the Chinese Pharmacopoeia (China, 2020). Rat blood and tissue homogenates (intestine and liver) were freshly collected and prepared. TMF, TAF, or TDF (5 μM) was incubated with each at 37°C for 0, 5, 15, 30, 60, 90, 120 min, and then the reaction was stopped and followed by LC-MS/MS analysis.

### 2.10 *In Situ* single-pass perfusion

To explore the intestinal permeability of different TFV prodrugs, an *in situ* single-pass perfusion experiment was carried out as we reported previously (Zhang et al., 2010). Briefly, an approximately 10-cm jejunum segment was isolated and Krebs-Ringer buffer containing 5 μM TMF, TAF, or TDF was perfused at a flow rate of 0.2 ml/min through the intestinal segment. Perfusate samples were collected every 15 min for 2 h from the outlet of the jejunum for LC-MS/MS analysis. The radius and length of the jejunum segment were measured at the end of the experiment. The intestinal permeability coefficient (P_eff_) was calculated as $P_{eff}=-\frac{Q_{in}}{A}\times \left(ln\frac{C_{out(corr)}}{C_{in}}\right)$ , where Q_in_ is the flow rate (mL/min); A is the surface area of the intestinal segment (cm^2^); C_in_ is the initial donor concentration (5 μM); C_out(corr)_ is the corrected concentration of perfusate samples after applying the weight correction factor: $C_{out\left(corr\right)}=C_{out}\times \frac{Q_{out}}{Q_{in}}$ .

### 2.11 Absorption forms of prodrug

18 male SD rats (*n* = 6) were anesthetized, fixed and administered 30 μmol/kg TMF, TAF or TDF orally. Blood (200 μl) was collected at 5, 30 min and 1 h post-dose from the rat hepatic portal vein (HPV) and inferior vena cava (IVC) for LC-MS/MS analysis.

### 2.12 LC-MS/MS analysis for prodrug and its metabolites

Since TMF, TAF, and TDF are all hydrolyzed into TFV and further phosphorylated into TFV-DP, an LC-MS/MS method was developed as we described previously (Ouyang et al., 2017) with minor modifications. All the analytes were detected in positive MRM mode, and the parent/daughter mass transitions for TMF, TAF, TDF, TFV, TFV-DP and IS (Lamivudine) were 491.2/346.0, 477.2/346.1, 520.2/270.1, 288.1/176.1, 477.2/346.1 and 230.3/112.0, respectively.

### 2.13 Statistical analysis

All the data are presented as the mean ± S.E. Two-tailed Student’s t-tests, one-way ANOVA, and nonparametric analysis were employed for statistical analyses. Differences were considered significant at *p < 0.05, **p < 0.01, and ***p < 0.001. Statistical data analysis was performed using GraphPad Prism.

## 3 Results

### 3.1 TFV ester prodrugs exhibited differential anti-HBV activity *in vitro* and *in vivo*

**FIGURE 2:**
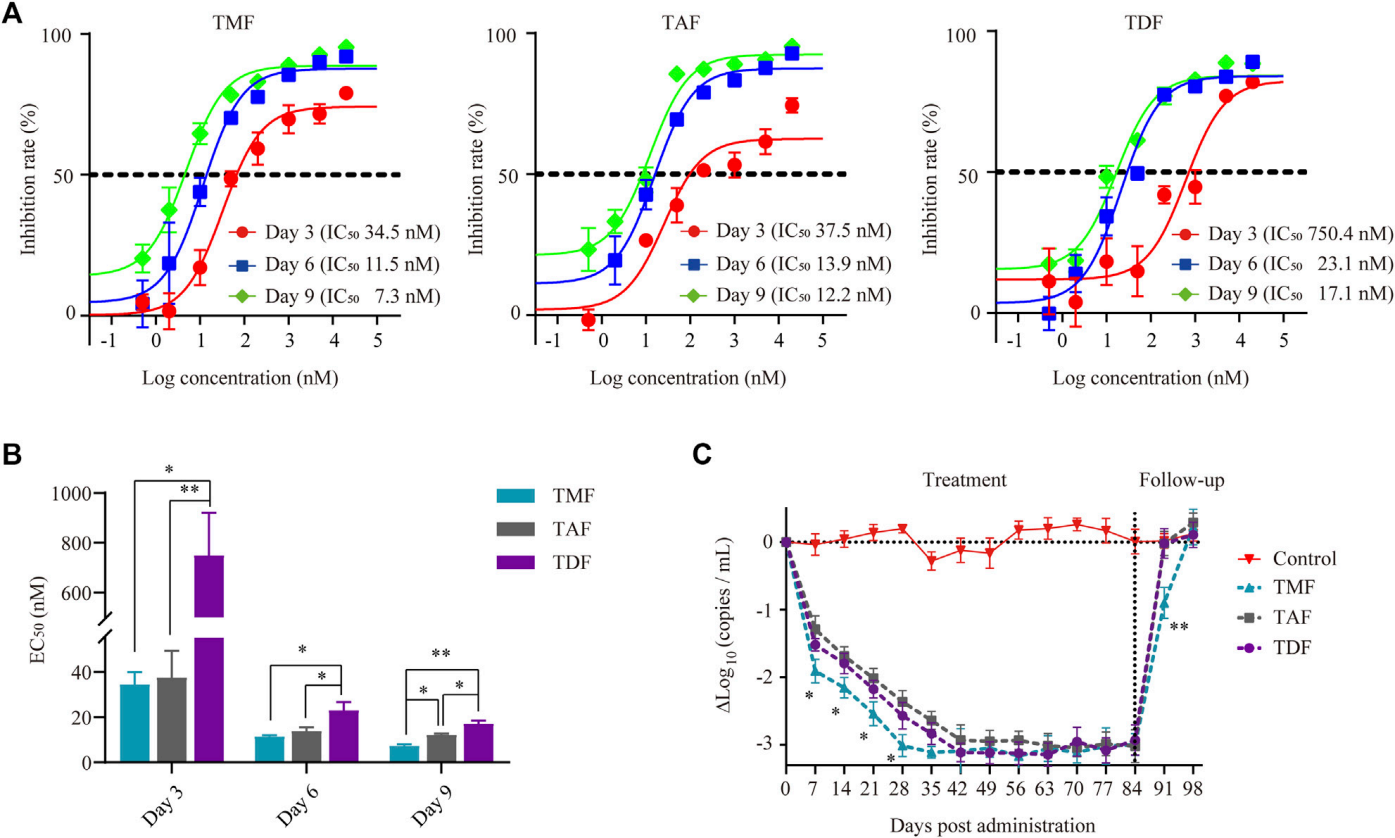
Anti-HBV activity of TFV prodrugs. **(A)** The inhibition rate of TMF, TAF and TDF on secreted HBV DNA in HepG2.2.15 cell culture supernatants after 3, 6, and 9 days of treatment, respectively (*n* = 5). **(B)** The EC_50_ value of each TFV prodrug after different treatment times. **(C)** Serum HBV DNA copies of HBV transgenic mice after treatment with normal saline (control group) or 60.7 μmol/kg TFV prodrugs for 84 days followed by a 14-daydrug withdrawal period (*n* = 8). *p < 0.05, **p < 0.01.

The HBV-positive hepatic cell line HepG2.2.15, which stably secretes HBV DNA into the supernatant medium, was used to compare the anti-HBV activities of different nucleotide analog(s) *in vitro*. With the extension of incubation time, the anti-HBV activity of each prodrug increased with decreased EC_50_ values (Figures 2A,B). TMF and TAF exhibited significantly stronger inhibition of HBV DNA replication than did TDF after 3, 6 or 9 days of treatment. In particular, the anti-HBV activity of TMF was significantly stronger than that of TDF and slightly stronger than that of TAF after 9 days of treatment, of which the EC_50_ value of TMF (7.29 ± 0.71 nM) was 2.34 times and 1.67 times smaller than that of TDF (17.09 ± 2.45 nM) and TAF (12.17 ± 0.56 nM), respectively.(*p* < 0.01 TMF vs. TDF; *p* < 0.05 TMF vs. TAF).

In HBV transgenic mice, all the drugs exhibited significant anti-HBV effects, as there was a sharp reduction in HBV DNA copies in serum to the same level after the first 42 days post-drug administration. The antiviral response of each prodrug was sustained until dosing discontinuance on day 84 due to the persistence of genomic HBV (Figure 2C). In particular, the serum HBV DNA-lowering effect of TMF seemed quicker than that of TAF and TDF, with a 0.5Δlog_10_ (copies/mL) difference (*p* < 0.05) during the HBV DNA decline period, which was approximately 16% of the maximal anti-HBV effect in this transgenic mouse model. Furthermore, when each prodrug was withdrawn (day 84), the rebound of serum HBV DNA in the TMF-treated group was the slowest.

### 3.2 TFV ester prodrugs rebalanced the abnormal hepatic metabolomics in HBV positive HepG2.2.15 cells to different extents

**FIGURE 3:**
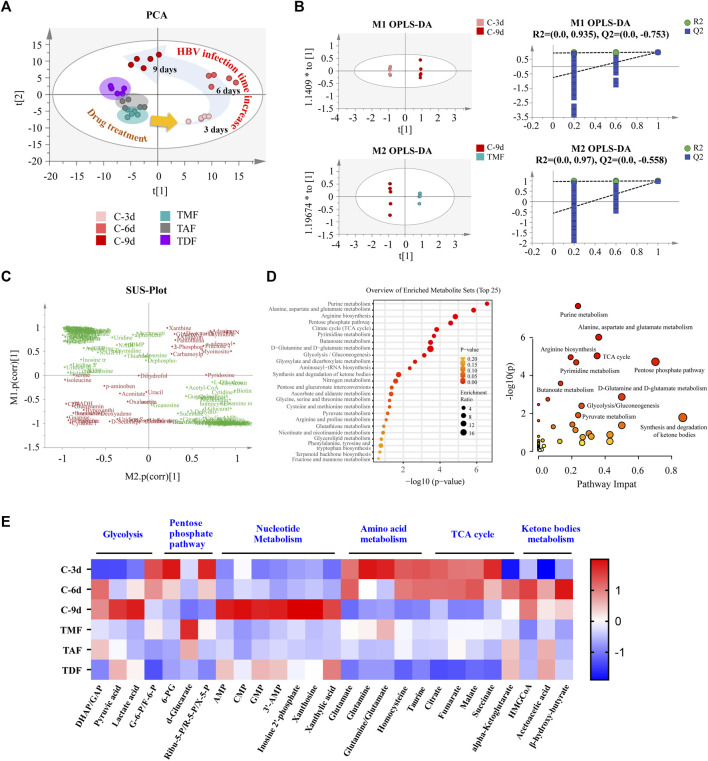
The rebalancing effect of TFV prodrugs on the abnormal metabolomics of HBV-positive HepG2.2.15 cells with continuous replication of HBV DNA. **(A)** Principal component analysis (PCA) was performed to reveal the intracellular metabolomics shift with HBV production and to compare the different callback effects of each TFV prodrug. **(B)** Orthogonal partial least squares-discriminant analysis (OPLS-DA) was performed to compare HBV DNA replication for 3 days vs. 9 days (upper), and 9 days *vs*. the TMF treatment group (bottom). **(C)** The shared part in **(B)** was combined and applied to shared and unique structure-plot analysis (SUS-plot) to determine the callback metabolites after TMF treatment. **(D)** Enrichment analysis and pathway analysis of the callback metabolites. **(E)** The main differential metabolites in HepG2.2.15 cells that changed with HBV DNA continuous replication after being corrected by each TFV prodrug were presented in a heatmap. The annotation labels C-3 days, C-6 days, C-9 days, TMF, TAF, and TDF indicated that HepG2.2.15 cells were cultured for 3, 6, and 9 days or were treated with TMF, TAF and TDF for 9 days, respectively. (DHAP: dihydroxyacetone phosphate; GAP: glyceraldehyde 3-phosphate; G-6-P: glucose 6-phosphate; F-6-p: fructose 6-phosphate; 6-PG: 6-phosphogluconate; Ribu-5-P: ribulose 5-phosphate; R-5-P: ribose-5-phosphate; X-5-P: xylulose 5-phosphate; AMP: adenosine monophosphate; CMP: cytidine monophosphate; GMP: guanosine monophosphate; 3′-AMP: 3′-adenosine monophosphate; HMGCoA: 3-hydroxy-3-methylglutaryl-CoA).

Along with the inhibition of HBV DNA replication, host intracellular biochemical metabolism was altered by TFV ester prodrugs. PCA analysis indicated that the metabolomic profiles of HepG2.2.15 cells were markedly shifted from the bottom-right direction to the upper-left direction along with the continuous replication of HBV DNA from 3–9 days. These shifts were corrected to different extents by TFV ester prodrugs, of which TMF was the most potent, while TDF was the weakest (Figure 3A). Next, based on OPLS-DA, SUS-plot and KEGG analysis, it was revealed that these call-back metabolites after TMF treatment were mainly enriched in the TCA cycle, glycolysis, pentose phosphate pathway, purine metabolism, pyrimidine metabolism, amino acid metabolism and ketone body metabolism (Figures 3B–D). Specifically, as shown in Figure 3E, glycolysis, nucleotide metabolism, and ketone body metabolism were upregulated, while pentose phosphate pathway, TCA cycle and amino acid metabolism were downregulated with HBV DNA production, and these changes were rebalanced by TFV ester prodrugs to different extents.

### 3.3 TFV ester prodrugs differentially corrected the disordered phospholipids in HBV-positive HepG2.2.15 cells

**FIGURE 4:**
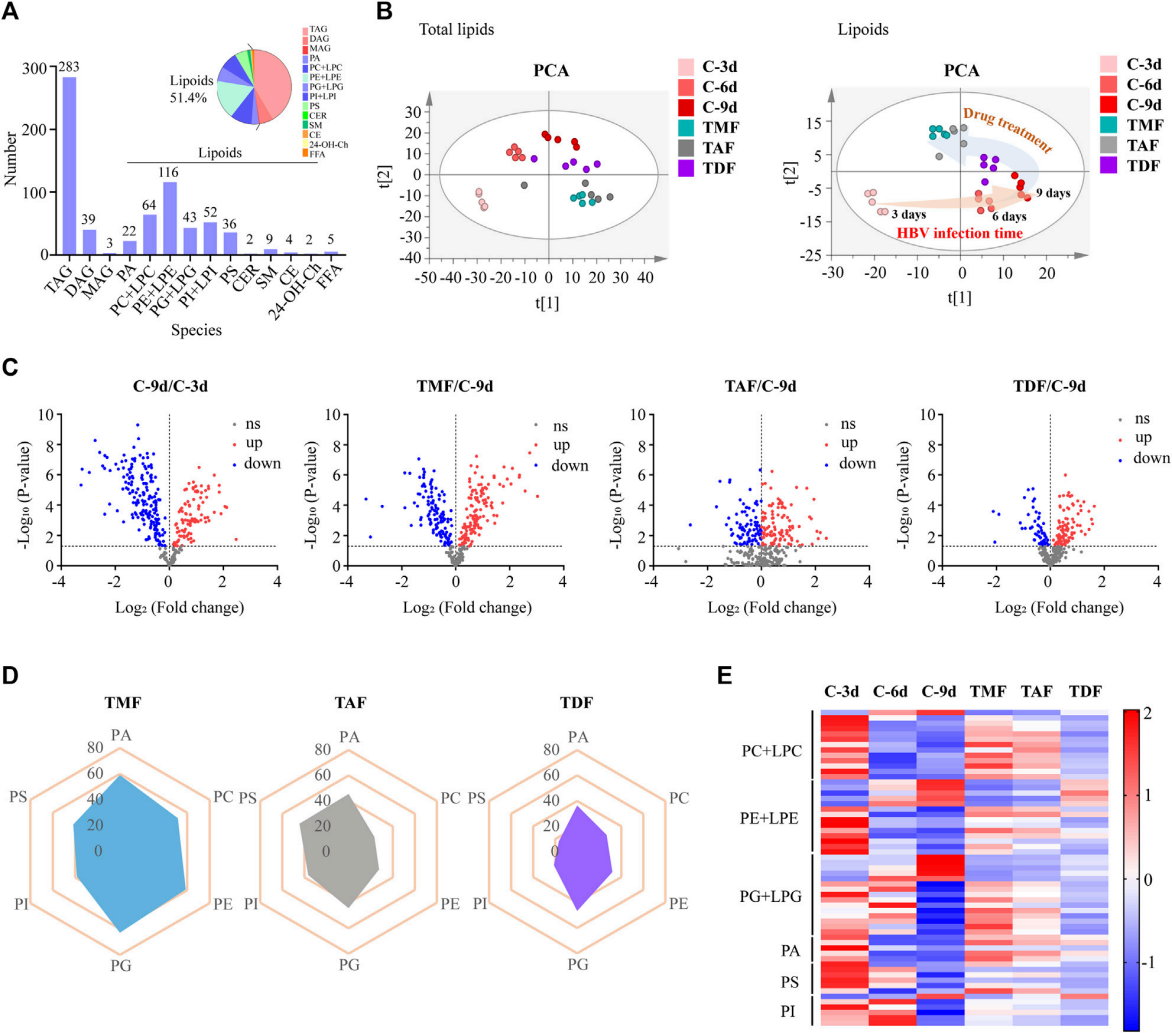
The correcting effect of TFV prodrugs on the abnormal lipidomics in HBV positive HepG2.2.15 cells with continuous replication of HBV DNA. **(A)** Numbers of lipids detected and percentage of different lipid species. **(B)** PCA was performed to exhibit the shift in total lipids (left) or lipoids (right) accompanied by HBV DNA replication time increase and to compare the different callback effects of each TFV prodrug. **(C)** Volcano plot of lipoids in HBV DNA replication for 3 days vs. 9 days and 9 days vs. each TFV prodrug treatment group. **(D)** Radar plot of the percentage of callback phospholipids in different TFV prodrug treatment groups. **(E)** Heatmap visualizing the intensities of the main differential phospholipids in different groups. (TAG: triacylglycerol; DAG: diacylglycerol; MAG: monoglyceride; PA: phosphatidic acid; PC: phosphatidylcholine; LPC: lysophosphatidylcholine; PE: phosphatidylethanolamine; LPE: lysophosphatidylethanolamine; PG: phosphatidylglycerol; LPG: lysoPhosphatidylglycerol; PI: phosphatidylinositol; LPI: lysophosphatidylinositol; PS: phosphatidylserine; CER: ceramide; SM: sphingomyelin; CE: cholesteryl ester; Ch: Cholesterol; 24-OH-Ch: 24-OH-Cholesterol; FFA: free fatty acid).

Furthermore, the callback efficacy of TFV ester prodrugs in lipids was investigated by lipidomics. A total of 680 lipids, including 283 triacylglycerols (TAGs), 39 diacylglycerols (DAGs), 3 monoglycerides (MAGs), 350 lipoids and 5 free fatty acids (FFAs), were detected in our method, and PCA analysis indicated that these prodrugs were more potently rebalanced in lipoids than in total lipids, so the subsequent analysis focused on lipoids (Figures 4A,B). From the volcano plot of host lipoids with HBV DNA replication for 3 days vs. 9 days and 9 days vs. each TFV ester prodrug treatment group, 104 lipoids, 112 lipoids, and 181 lipoids were found to be rebalanced by TDF, TAF and TMF, respectively (Figure 4C). The most significantly corrected lipoids were glycerophospholipids. In particular, TMF was considered the most prominent in terms of both the amount and the degree of the callback glycerophospholipids, followed by TAF, while TDF still exhibited the weakest correcting effect (Figures 4D,E).

### 3.4 TMF produced more active metabolite TFV-DP in hepatic cells

**FIGURE 5:**
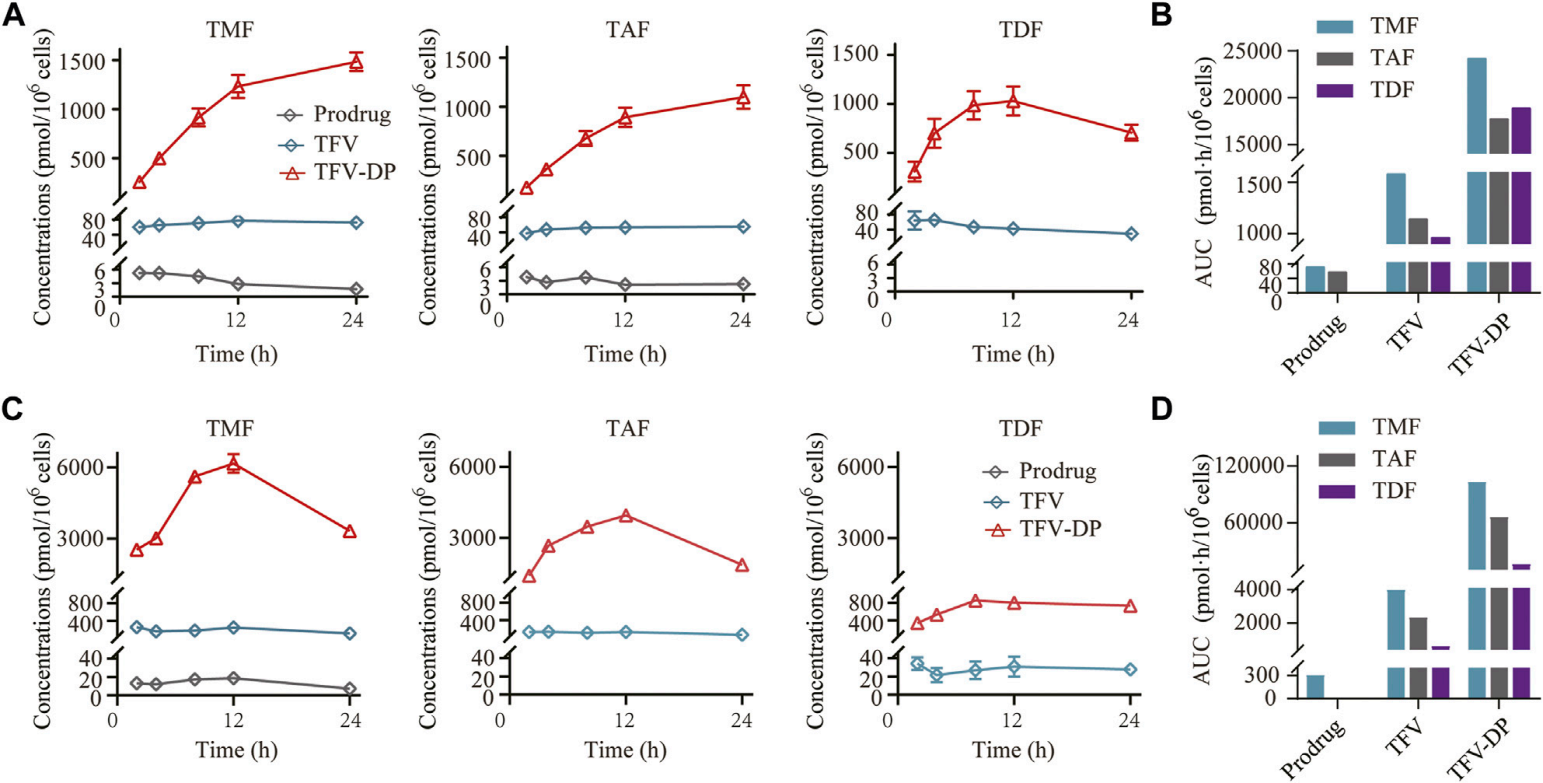
Targeted cell activation of TFV prodrugs. **(A)** Intracellular concentration-time profiles and **(B)** intracellular drug exposure of prodrug, TFV and TFV-DP after incubating 5 μM of each TFV prodrug with HepG2.2.15 cells (*n* = 3). **(C)** Intracellular concentration-time profiles and **(D)** intracellular drug exposure of prodrug, TFV and TFV-DP after incubating 5 μM of each TFV prodrug with primary rat hepatocytes (*n* = 3).

As shown in Figure 5A, all the prodrugs were efficiently phosphorylated to TFV-DP in HBV positive HepG2.2.15 cells. However, the three prodrugs exhibited different patterns: TMF and TAF provided persistent increases in TFV-DP levels throughout 24 h, while TDF reached peak value at 12 h post-drug treatment. Similar to HepG2.2.15 cells, TFV-DP was also the main form in primary rat hepatocytes after TFV ester prodrugs treatment. Moreover, only TMF could be detected as an ester form within primary rat hepatocytes among these prodrugs (Figure 5C). In particular, in both HepG2.2.15 cells and primary rat hepatocytes, TMF provided the highest TFV-DP level and exposure, followed by TAF and TDF (Figures 5B,D), which was consistent with their abovementioned pharmacological potency.

### 3.5 TMF gained advantages in liver enrichment and metabolic activation in rats

**FIGURE 6:**
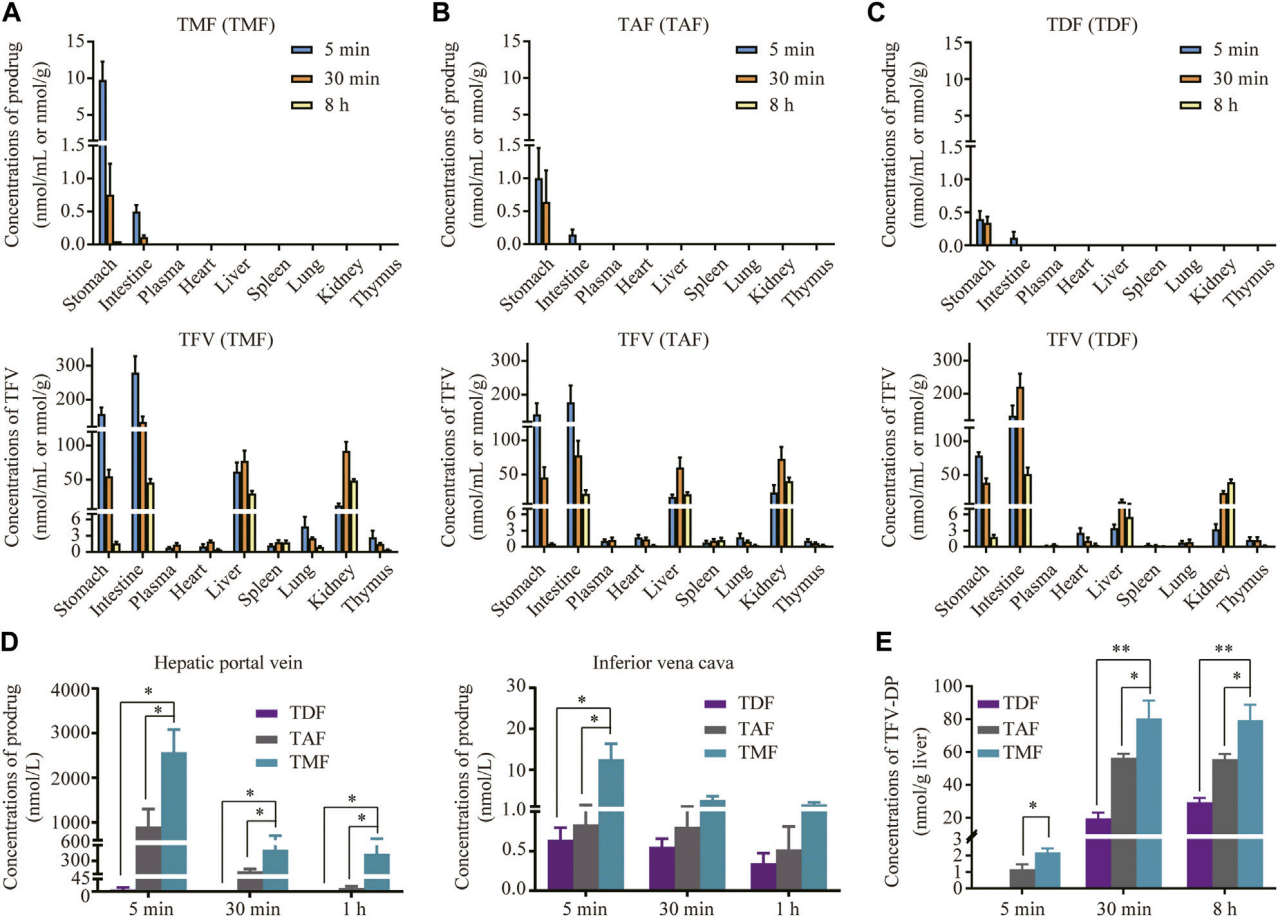
Targeted distribution and activation of TFV prodrugs. **(A–C)** Tissue distribution of each prodrug in SD rats orally administered TMF, TAF or TDF at 30 μmol/kg at 5, 30 min, and 8 h (*n* = 6). **(D)** Concentration of TFV prodrugs in rat hepatic portal vein or inferior vena cava after oral administration with each TFV prodrug at 30 μmol/kg at 5, 30 min, and 1 h (*n* = 6). **(E)** TFV-DP levels in rat liver after oral administration of 30 μmol/kg TMF, TAF or TDF at 5, 30 min, and 8 h (*n* = 6). *p < 0.05, **p < 0.01.

As shown in Figures 6A–C, TMF, TAF and TDF could not be detected in the ester form in any of the tissues except the stomach and intestine after oral administration to rats. Their hydrolyzed metabolite TFV was distributed widely after prodrugs administration, and the TFV level in the liver after TMF administration was significantly higher than that after TAF or TDF administration (TMF > TAF > TDF). Furthermore, the level of intact TMF in the ester form in rat HPV reached 2097.5 nM at 5 min after drug administration, which was 279.3-fold and 2.3-fold that of TDF and TAF, respectively (Figure 6D). In contrast, all the prodrugs decreased sharply in IVC in the ester form, and only 10.5 nM TMF could be detected at 5 min after drug administration. Nevertheless, the level of intact TMF in the ester form was still significantly higher than that of TAF and TDF (Figure 6D). The subtraction of each prodrug between HPV and IVC was due to hepatic uptake and metabolism. As shown in Figure 6E, the antiviral active metabolite TFV-DP could be detected in the liver just 5 min after TMF and TAF administration, but not after TDF administration. Similar to the TFV level in the liver, TMF and TAF also provided 3.55 times and 2.48 times higher exposure of TFV-DP in the liver than did TDF, respectively. Furthermore, TMF provided a slightly higher exposure of TFV-DP than did TAF, which was 1.43-fold that of TAF.

### 3.6 TMF possessed much higher bioavailability in preclinical animals

**FIGURE 7:**
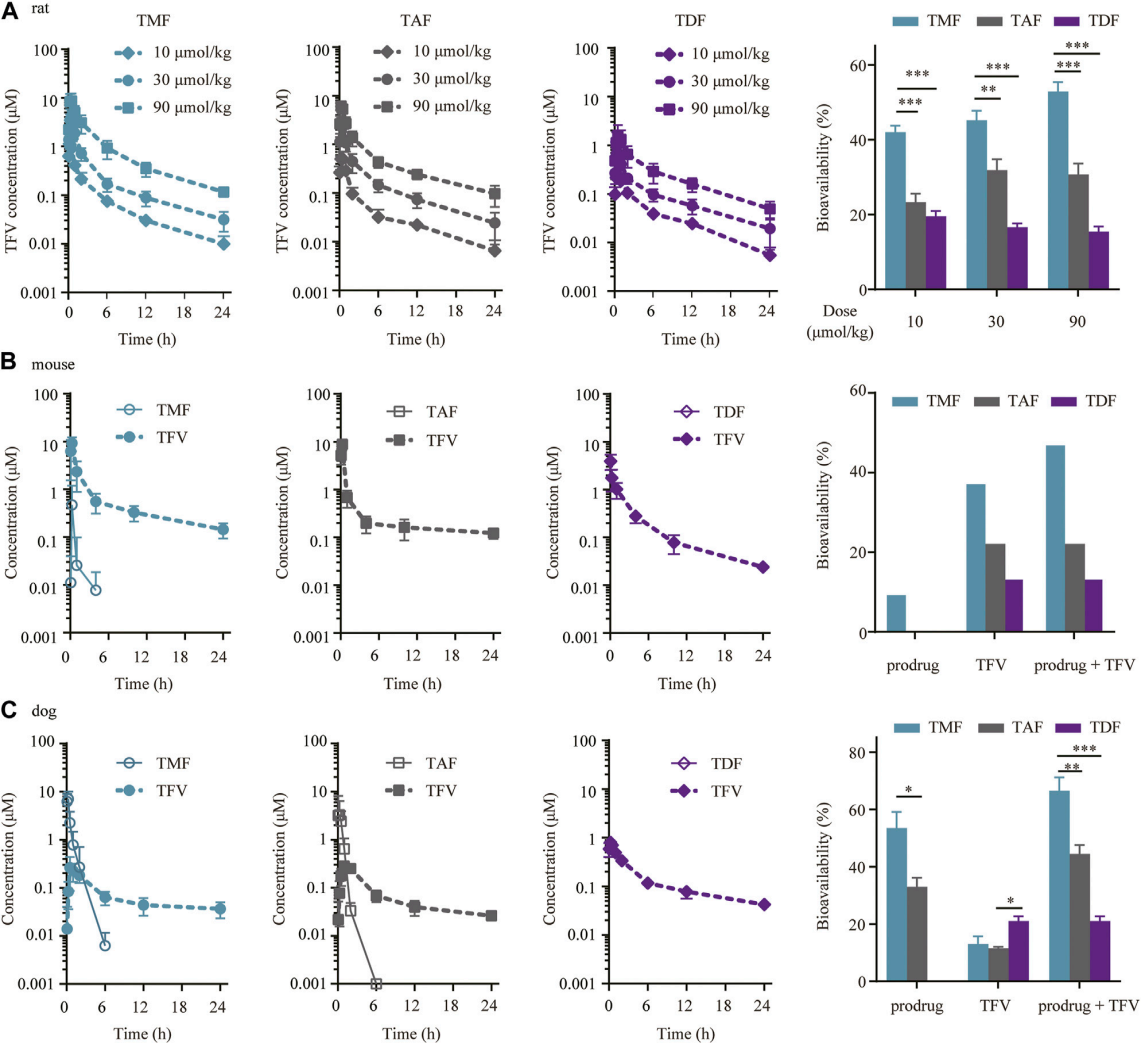
Preclinical plasma pharmacokinetics of TFV prodrugs. **(A)** Plasma concentration-time profiles and bioavailabilities of each TFV prodrug in SD rats orally administered 10, 30 or 90 μmol/kg TMF, TAF or TDF (*n* = 6). **(B)** Plasma concentration-time profiles and bioavailabilities of each TFV prodrug in C57BL/6 mice after oral administration of 60.7 μmol/kg TMF, TAF or TDF (*n* = 6). **(C)** Plasma concentration-time profiles and bioavailabilities of each TFV prodrug in beagle dogs following oral administration of 16.3 μmol/kg TMF, TAF or TDF (*n* = 4). *p < 0.05, **p < 0.01, ***p < 0.001.

In SD rats, all three prodrugs (TMF, TAF and TDF) were so rapidly hydrolyzed into TFV that the prodrug form could not be detected in plasma. The TFV levels and the exposure of TFV in plasma for each dosage group of the same prodrug were presented in a dose dependent manner. Furthermore, the values of t_max_ and t_1/2_ of TFV for each prodrug were similar regardless of the dose (Figure 7A and Table 1). The characteristics of the above pharmacokinetic parameters indicated that a linear pharmacokinetics of TFV prodrugs in rats. The absolute bioavailability of TFV after TMF administration (46.70% ± 5.59%) was significantly higher than that of TDF (17.21% ± 2.09%) or TAF (28.60% ± 4.65%), with approximately 2.71- and 1.63 -fold increases (Table 1).

**TABLE 1:** Preclinical pharmacokinetic parameters of TMF, TAF and TDF after oral administration^a^.

Drug	Dose	Analyte	C_max_	T_max_	t_1/2_	AUC_0-t_	AUC_0-∞_	F(%)^c^
	(μmol/kg)		(μM)	(h)	(h)	(μM·h)	(μM·h)	
Rat ^b^								
TMF	10	TFV	1.20 ± 0.51	0.32 ± 0.15	7.26 ± 3.97	2.04 ± 0.38	2.16 ± 0.36	42.00 ± 6.93
	30		3.99 ± 2.27	0.40 ± 0.18	7.87 ± 2.30	6.59 ± 1.53	6.97 ± 1.62	45.22 ± 10.49
	90		9.59 ± 3.47	0.44 ± 0.24	6.77 ± 1.86	23.30 ± 4.96	24.46 ± 4.72	52.88 ± 10.21
TAF	10	TFV	0.66 ± 0.27	0.46 ± 0.25	7.54 ± 2.86	1.12 ± 0.27	1.20 ± 0.29	23.28 ± 5.67
	30		2.60 ± 1.04	0.31 ± 0.15	7.88 ± 3.37	4.57 ± 1.26	4.91 ± 1.36	31.85 ± 8.84
	90		6.53 ± 1.16	0.30 ± 0.11	7.87 ± 1.83	13.02 ± 2.30	14.20 ± 3.01	30.69 ± 6.50
TDF	10	TFV	0.27 ± 0.07	0.50 ± 0.16	6.03 ± 2.08	0.95 ± 0.19	1.00 ± 0.18	19.52 ± 3.48
	30		0.72 ± 0.21	0.39 ± 0.18	8.39 ± 4.99	2.29 ± 0.46	2.57 ± 0.44	16.65 ± 2.86
	90		1.81 ± 1.01	0.63 ± 0.17	7.48 ± 3.25	6.54 ± 2.16	7.15 ± 2.18	15.46 ± 4.71
Mouse								
TMF	60.7	TMF	0.47	0.25	1.73	0.28	0.30	9.70
		TFV	9.20	0.25	6.78	16.26	17.67	37.56
TAF	60.7	TAF	BLQ	NC	NC	NC	NC	NC
		TFV	8.80	0.25	4.88	9.30	10.15	22.59
TDF	60.7	TDF	BLQ^d^	NC	NC	NC	NC	NC
		TFV	3.95	0.08	4.62	5.39	5.55	13.55
Dog								
TMF	16.3	TMF	7.89 ± 3.04	0.17 ± 0.10	0.97 ± 0.15	3.68 ± 2.20	3.69 ± 2.21	53.49 ± 11.34
		TFV	0.29 ± 0.15	0.94 ± 0.72	13.90 ± 3.06	1.59 ± 0.59	2.35 ± 0.98	13.03 ± 5.46
TAF	16.3	TAF	5.12 ± 3.68	0.27 ± 0.17	0.51 ± 0.31	2.35 ± 1.20	2.35 ± 1.20	32.96 ± 6.46
		TFV	0.28 ± 0.05	1.25 ± 0.50	12.67 ± 3.09	1.60 ± 0.25	2.07 ± 0.20	11.51 ± 1.10
TDF	16.3	TDF	BLQ	NC	NC	NC	NC	NC
		TFV	0.81 ± 0.22	0.31 ± 0.13	12.70 ± 2.21	2.99 ± 0.36	3.79 ± 0.59	21.02 ± 3.29

a Data are expressed as the mean value of 6 rats, 6 mice or 4 dogs.

b Only TFV were detectable after oral administrations of TMF, TDF or TAF in rats.

c The bioavailability (F) of TFV was calculated by comparing the resulting plasma TFV AUC_0-∞_ to that observed after i.v. administration of TFV itself; the bioavailability of prodrug was calculated by comparing the resulting plasma prodrug AUC_0-∞_ to that observed after i.v. administration of corresponding prodrugs.

d BLQ, below limit of quantification; NC, not calculated.

In C57BL/6 mice, only TMF itself could be detected in plasma within 4 h after administration, with a T_max_ of 0.25 h, a t_1/2_ of 1.73 h and a bioavailability of 9.70%, while neither TAF nor TDF itself could be detected in plasma. Meanwhile, the AUC of TFV in plasma showed a striking difference among the three TFV ester prodrugs, in which the AUC of the TMF group (16.26 μM h) was 3.01 or 1.75 times that of the TDF (5.39 μM h) and TAF (9.30 μM h) groups. Therefore, the total bioavailability of these prodrugs was TMF (47.26%) > TAF (22.59%) > TDF (13.55%), of which the total bioavailability included both ester and TFV forms for TMF but only TFV forms for TAF and TDF (Figure 7B, Table 1).

The pharmacokinetic profiles of each prodrug in beagle dogs were quite similar to those in C57BL/6 mice. After drug administration, TMF and TAF were detected in plasma as the prodrug forms. TMF maintained longer (t_1/2_ 0.97 ± 0.15 h vs. 0.51 ± 0.31 h) and exposed higher (AUC_0-t_ 3.68 ± 2.20 μM h vs*.* 2.35 ± 1.20 μM h) in the ester form in plasma than did TAF, and thus led to the bioavailability difference between TMF and TAF (53.49% ± 11.34% vs*.* 32.96% ± 6.46%) (Table 1). For the TFV form in plasma, the bioavailability in dogs was 13.03% ± 5.46%, 11.51% ± 1.10%, and 21.02% ± 3.29% for TMF, TAF, and TDF, respectively (Figure 7C). Hence, the total bioavailabilities of TMF, TAF and TDF, including both ester and TFV forms, were 66.52%, 44.47%, and 21.02%, among which TMF ranked the highest.

### 3.7 Intestinal stability contributed to better absorption of TMF in the ester form

To explain the absorption differences among TMF, TDF, and TAF, potential steps involved in prodrug absorption, including gastrointestinal fluid stability, intestinal permeability and first pass effect, were evaluated.

**FIGURE 8:**
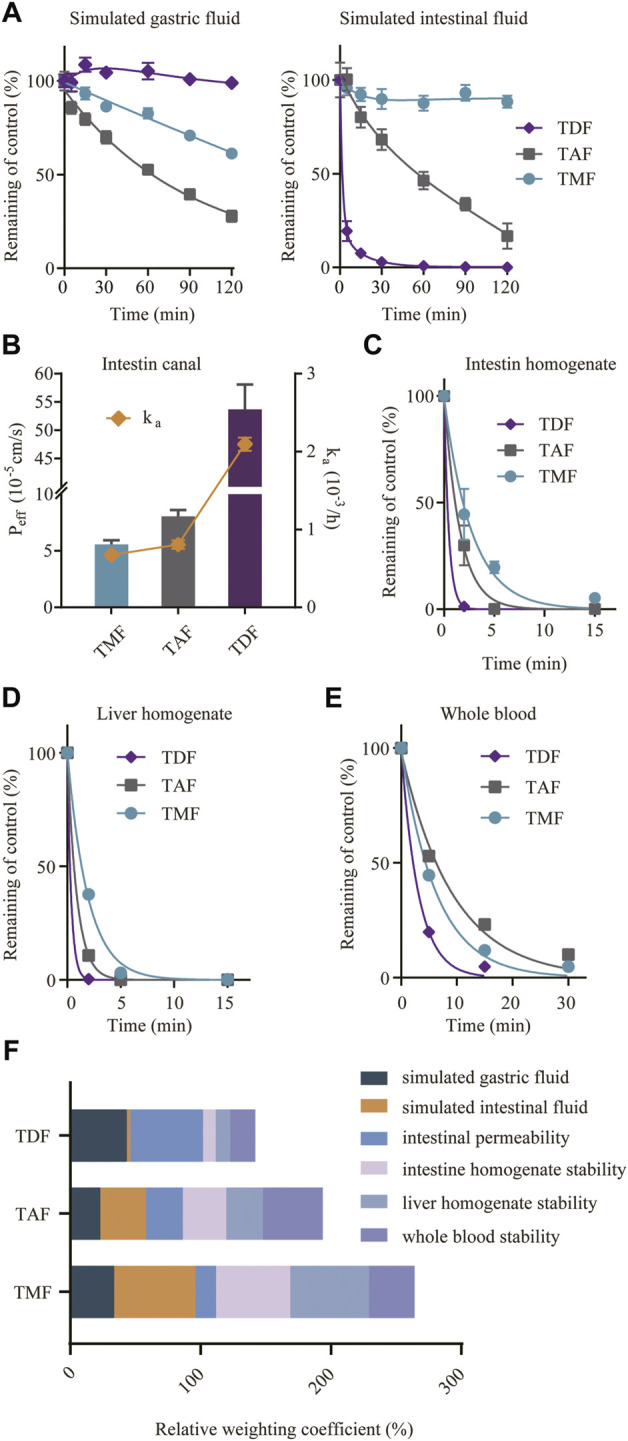
Factors involved in the absorption of TFV prodrugs. **(A)** Stability of TFV prodrugs in simulated gastric fluid or simulated intestinal fluid. **(B)** Intestinal permeability of TFV prodrugs *in situ* after single-pass intestinal perfusion of the rat jejunum. Stability of TFV prodrugs in fresh rat intestine homogenate **(C)**, liver homogenate **(D)** and whole blood **(E)**. **(F)** Relative weighting coefficient of each TFV prodrug for the abovementioned factors.

As shown in Figure 8A, TDF was stable in SGF but degraded rapidly in SIF with a short t_1/2_ of 13.7 min; TAF showed higher stability in SIF with an elimination t_1/2_ of 48.4 min but a higher degradation in SGF than TDF (the degradation t_1/2_ of TAF in SGF was 71.3 min). Comparatively, TMF was extremely stable in SIF with a t_1/2_ over 120 min and relatively stable in SGF.

An *in situ* single-pass perfusion experiment on rat jejunum (Figure 8B) revealed that TDF possessed higher permeability (P_eff_ 53.7 × 10^−5^ cm/s) than TAF (P_eff_ 8.1 × 10^−5^ cm/s) or TMF (P_eff_ 5.6 × 10^−5^ cm/s). The absorption rate constants (k_a_) of TMF, TAF and TDF were 0.675 × 10^−3^/h, 0.807 × 10^−3^/h and 2.095 × 10^−3^/h, respectively.

For the first-pass effect, the prodrug stabilities in intestine/liver homogenate and blood were assessed. As shown in Figures 8C–E, all the prodrugs were eliminated rapidly in these matrices. In particular, TDF degraded the fastest in all the extracts, with an elimination t_1/2_ of 3.58 min in rat blood, and approximately 0.3 min in rat intestine and in liver homogenates (0.32 and 0.36 min). TMF was more stable than TAF in rat intestine and in liver homogenate (t_1/2_ 3.80 min vs*.* 1.59 min in intestine homogenates; t_1/2_ 1.72 min vs*.* 1.06 min in liver homogenates), despite a higher degradation in blood (t_1/2_ 6.97 min vs*.* 9.31 min). As mentioned above, the absorption of the TFV ester prodrug was related to many factors; therefore, the normalized weighting coefficient, defined as the ratio of the AUC or P_eff_ of each prodrug to the sum of the AUC or P_eff_ of the three prodrugs, was calculated to evaluate the absorption of the TFV ester prodrug from an overall perspective. Figure 8F, the sum of the weighting coefficients of TMF was highest, which was consistent with the highest bioavailability *in vivo*. Furthermore, among different factors, intestinal stability, including the intestinal lumen and intestinal epithelium, was the main factor affecting the bioavailability of the TFV ester prodrug *in vivo*.

## 4 Discussion

Current treatment of HBV infection relies on NAs and IFNs, and NAs are superior due to their effective viral suppression, good-tolerance and favorable pharmacokinetics (Smolders et al., 2020). TFV is a potent and very tolerant NA against HBV (Balzarini et al., 1993; Heijtink et al., 1994; Deeks et al., 1998; Michailidis et al., 2012; Li et al., 2013), but it displays poor bioavailability (Cundy et al., 1998), poor cell permeability (Lee and Martin, 2006; Durand-Gasselin et al., 2009) and even nephrotoxicity (Giesler et al., 2016). As prodrug design could improve pharmacokinetics, drug delivery and drug efficacy (Jana et al., 2010; Yu et al., 2016), a series of TFV prodrugs were designed and synthesized (Pradere et al., 2014; Wang et al., 2019). TDF, TAF and TMF are three ester prodrugs of TFV that have been approved by the FDA in the US or the NMPA in China for HBV therapy. In this work, the above-mentioned three TFV ester prodrugs were used as probes to explore the key factors in the pharmacokinetics process that influenced their anti-HBV activity and hepatic biochemical metabolism regulating effect, which will benefit new drug design, modification and evaluation in the future.

First, the anti-HBV activities of the three TFV prodrugs were evaluated *in vitro* based on HBV-positive HepG2.2.15 cells, which can secrete HBV DNA particles. TMF displayed 2.3-fold higher antiviral activity than TDF and 1.6-fold better efficacy than TAF after 9 days of treatment (Figures 2A,B). The difference of EC_50_ might not be that big, but it does exist. The purpose of this paper is not to highlight the advantages of a certain drug, but to find the correlation among the structure, pharmacokinetics and activity of different TFV ester prodrugs. Therefore, even if they are not extremely significant differences, these differences can also reflect the impact of structure on pharmacokinetics and efficacy, or put forward a trend direction for future structure modification, And this result could be attributed to the higher intracellular TFV-DP level of TMF than that of TDF or TAF (Figure 5). Because TFV is an acyclic nucleotide analog, it should undergo intracellular phosphorylation to form TFV-DP in hepatocytes, which competitively prevents endogenous 2′-deoxyadenosine triphosphate from incorporation by the viral reverse transcriptase and causes subsequent chain-termination of viral DNA replication. TMF produced the most TFV-DP in hepatocytes and thus exhibited the most potent HBV inhibition effect. Therefore, the TFV-DP level in hepatocytes produced by the TFV prodrugs played a key role in HBV inhibition.

Such differential anti-HBV effects of these TFV prodrugs were also observed directly in the treatment of HBV transgenic mice *in vivo*. Although these three TFV prodrugs finally achieved the same anti-HBV effect after 42 days treatments, their effects in the HBV decline period were not in complete accord, of which the anti-HBV efficacy of TMF was better than that of TDF or TAF. TMF successively decreased serum HBV DNA copies throughout 28 days post-drug administration with a drop of 3.03 Δlog_10_ (copies/mL) from the baseline (Figure 2C), and the HBV DNA level was sustained until dosing discontinuance on day 84 due to the persistence of genomic HBV. Moreover, TDF/TAF showed a decrease of 2.53 Δlog_10_ (copies/mL) on the 28th day and achieved 3.1 Δlog_10_ (copies/mL) for a 1–2 weeks delay. Such a 0.5 Δlog_10_ (copies/mL) lower difference (approximately 16% of the maximal anti-HBV effect in such a transgenic mouse model) between TMF and TAF/TDF is analogous to the improvement from entecavir to TDF with 0.2 Δlog_10_ (copies/mL) (Park et al., 2017; Wan et al., 2019), which suggested a substantial advance in the structure of TMF. When the three prodrugs were withdrawn, TMF-treated HBV transgenic mice also exhibited a slower bounce to baseline levels (14 days) than did TDF/TAF mice (7 days). These observations (TMF>TAF ≈ TDF) were a little different from *in vitro* results (TMF > TAF > TDF). This might be related to species differences, because *in vitro* experiments were carried out with human derived cells, while *in vivo* experiments were carried out on mice. Meanwhile, the *in vivo* anti-HBV effects of the three TFV prodrugs in mice were quite correlated with their *in vivo* exposure in mice, as the AUC of TDF was close to that of TAF in mice, and both of them were much smaller than that of TMF in mice (Figure 7B).

Considering that HBV DNA continuous replication can cause steatohepatitis, fibrosis, and even hepatocellular carcinoma, all of which are generally called metabolic diseases, metabolomics and lipidomics analysis of host hepatic cells with or without TFV prodrugs treatments were performed. With HBV replication (9 days vs. 3 days), glycolysis, nucleotide metabolism and ketone body metabolism were upregulated, while pentose phosphate pathway, TCA cycle and amino acid metabolism were downregulated (Figure 3). Wan, Q (Wan et al., 2017) found that glucose in HBV-infected hepatocytes was used more for glycolysis and pentose phosphate pathway to synthesize the precursors of macromolecules and nucleotides that are required for virus replication but was rarely used for the TCA cycle. Sadrolodabaee, L. (Sadrolodabaee et al., 2013) used proteomics analysis and found that multiple enzymes in the glycolysis pathway were upregulated in HepG2.2.15 cells. Another proteomics study suggested that three key TCA cycle-related enzymes (*Idh3a*, *Dlst*, *Suclg2*) were downregulated in HBV-transgenic mice (Ding et al., 2009). Taken together, our results are almost in full accordance with these reports except for the downregulated pentose phosphate pathway metabolites. We speculated that HBV might hijack the metabolites of pentose phosphate pathway to produce massive nucleotides to satisfy its continuous virus replication. Meanwhile, metabolic disorders were found in intracellular lipids, especially glycerophospholipids (Figure 4). HBV has been reported to hijack the glycerol-3-phosphate-NADH shuttle, leading to reduced glycerophospholipid and increased plasmalogen species (Schoeman et al., 2016), and the latter has been revealed as the preferred lipid species in the HBV envelope and surface antigen particles (Satoh et al., 1990). On this basis, three TFV prodrugs were found to rebalance the abovementioned disorders of hepatic biochemical metabolism to different extents with HBV replication inhibition, which is also considered to be tightly related to TFV-DP levels in hepatocytes.

Since the regulatory effect of TMF on HBV positive hepatic metabolism was considered to be mediated by HBV inhibition through TFV-DP, the superiority of TMF in liver enrichment and metabolic activation was elucidated. The tissue distribution of each TFV prodrug was determined and compared in rats. TMF, TAF, and TDF were detected only in the stomach and the intestine, and their released TFV was distributed widely after being absorbed into circulation and mainly accumulated in the liver and kidney (Figures 6A–C). The great difference in TFV ester levels between HPV and IVC (Figure 6D) indicated that TMF not only crossed the intestinal barrier mainly in an intact ester form, but also experienced hepatic metabolism. Hence, we further compared the TFV-DP levels in the livers of rats after treatment with different prodrugs at the same dose. Among the three prodrugs, TMF provided 1.43-fold and 3.55-fold higher liver levels of TFV-DP in rats than did TAF and TDF (Figure 6E), which indicated that TMF could promote more accumulation of active metabolites in disease-targeting tissue, and possibly explained its better anti-HBV potency and hepatic metabolism regulating effect over TDF or TAF.

Bioavailability improvement might be an important assurance of liver enrichment. Hence, the bioavailabilities of TMF, TAF and TDF were compared in various preclinical animals, including SD rats, C57BL/6 mice and beagle dogs. All the three prodrugs effectively delivered TFV to the system circulation (Figure 7), and TMF provided the highest bioavailability in all the species. In particular, TMF produced a much higher plasma concentration as an intact prodrug in mice and dogs (Figures 7B,C), indicating more effective cell loading of TMF than of TDF or TAF (Babusis et al., 2013). According to previous research, the higher stability of TAF in plasma guarantees its better target loading and higher antiviral efficacy than TDF (Babusis et al., 2013; Murakami et al., 2015). As a result, a 1/10 dose of TAF provides comparable antiviral activity to TDF in clinical applications with less toxicity (Ruane et al., 2013). Accordingly, the newly designed TMF was maintained longer in dog/mouse plasma in the ester form and provided higher plasma exposure than TAF, indicating the potential safety and efficacy advantages of TMF in clinical treatment.

Subsequently, the possible mechanisms that influence the bioavailability of TFV ester prodrugs were analyzed. The main factors for oral drug absorption into systemic circulation include stomach fluid, intestinal fluid, intestinal barrier, liver and blood. Therefore, gastrointestinal fluid stability was analyzed first. Although the three prodrugs are similar in structure, different moieties contribute to the distinct stability behaviors of TFV prodrugs. TDF was the most stable in SGF but the most unstable in SIF; moreover, TAF was more stable in SIF than in SGF (Golla et al., 2016). TMF was proven to be stable in both SGF and SIF (Figure 8A). Higher gastrointestinal stability, particularly greater stability in SIF, guaranteed persistent intestinal absorption of intact TMF, as drugs were mainly absorbed from the small intestine (Murakami, 2017). Thus, after gastrointestinal fluid degradation, TMF possessed the most remaining amount of intact prodrug. Subsequently, prodrugs need to penetrate the intestinal barrier, and the permeability of each prodrug across the jejunum was assayed by single-pass perfusion. Although it was surprising to find that the permeability order was TDF > TAF > TMF, the P_eff_ values of all three prodrugs were at the level of 10^−5^ cm/s, which indicated high permeability (Dubbelboer et al., 2019) (Figure 8B). Therefore, it was believed that permeability was not the restrictive factor for TFV prodrug absorption. However, when the TFV prodrug penetrated into the intestinal tissue, TDF was degraded the most rapidly, while TMF was degraded the slowest among the three (Figure 8C). The oral bioavailability of TFV ester prodrugs was speculated to be correlated with their *in vitro* stability in intestinal homogenate (Shaw et al., 1997). When the remaining prodrug moved to the liver, TDF was further degraded, while TMF was again significantly more stable than TAF and TDF (Figure 8D). After first pass at the intestine and liver, the remaining TFV prodrug was delivered to the blood circulation, and TMF/TAF was much more stable than TDF in the blood (Figure 8E). Considering the abovementioned factors, stability in intestinal fluid determines the actual amount of TFV prodrug at the absorption site, and hepatic/intestinal stability determines the amount of maintained prodrug in circulation. Therefore, the oral bioavailability of TFV prodrugs mainly depends on their comprehensive stability at the absorption site, especially in the intestine. However, certain species differences in the pharmacokinetic behaviors of TFV ester prodrugs might exist, and our results are limited in preclinical animal models. Currently, we are attempting to establish a mathematical model to better describe and predict the relationship between the structures and pharmacokinetic behaviors of different TFV ester prodrugs in human.

## 5 Conclusion

**FIGURE 9:**
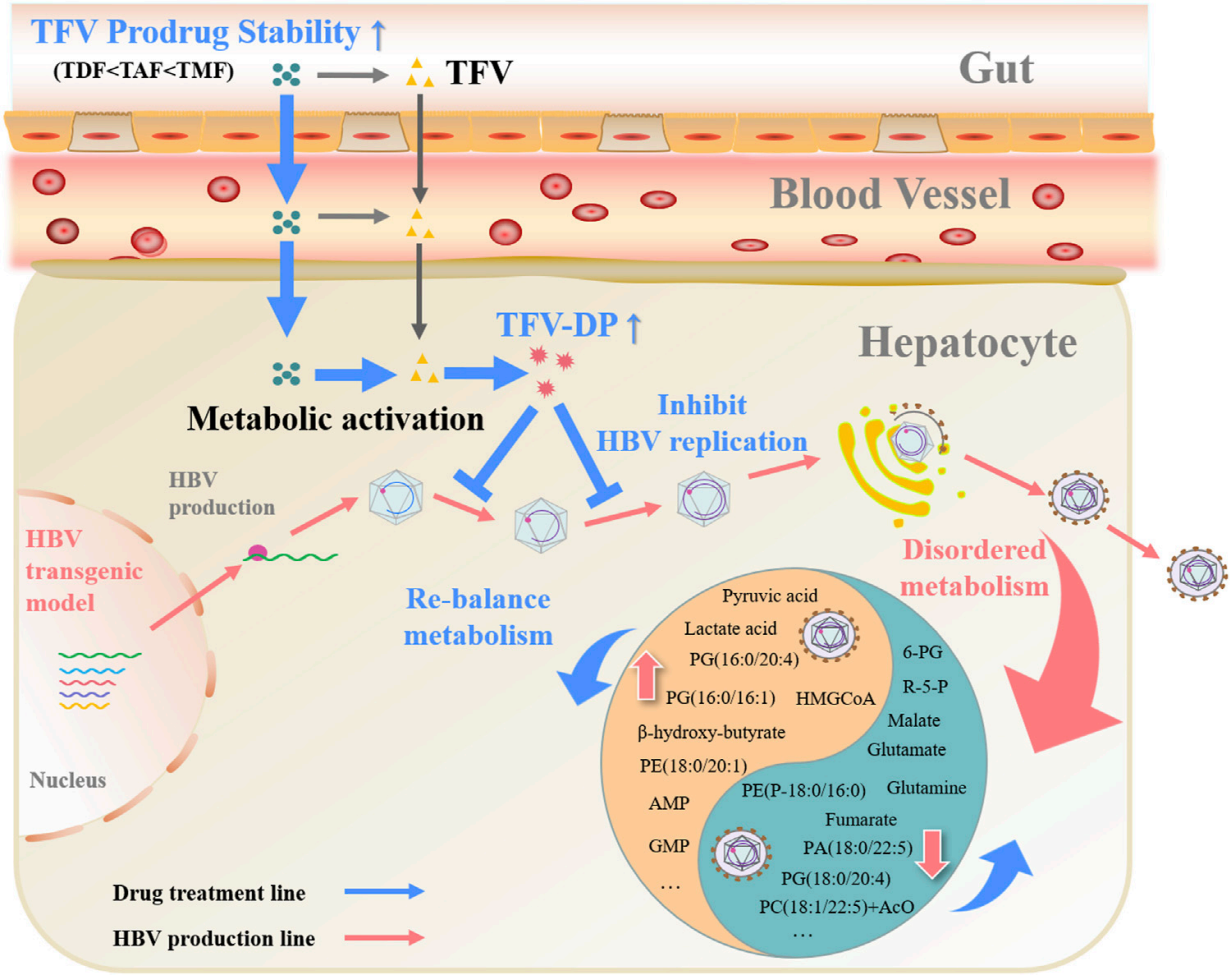
Graphic abstract of TFV ester prodrugs: relationship between pharmacokinetics and efficacy.

In our study, the antiviral activity, hepatic biochemical metabolism regulating effect, targeted distribution and metabolic activation, bioavailability, gastrointestinal fluid stability, intestinal permeability and first-pass effect of three currently marketed TFV ester prodrugs were evaluated and compared. TMF and TAF exhibited significantly stronger anti-HBV efficacy and correcting effects for disordered hepatic biochemical metabolism than TDF *in vitro* and *in vivo*, while TMF was slightly superior to TAF. These different pharmacological activities of TFV prodrugs are believed to be attributed to their differences in pharmacokinetic characteristics: the greater intestinal stability and bioavailability of TFV prodrug in preclinical animals provide better target loading, especially higher hepatocytes level of pharmacologically active TFV-DP, which is tightly related with the efficacy of such prodrug (Figure 9). Therefore, our research provides new insights and a basis for the design, modification and evaluation of TFV prodrugs in the future.

## Data Availability

The raw data supporting the conclusions of this article will be made available by the authors, without undue reservation.

## Abbreviations

TFV, tenofovir; NAs, Nucleos(t)ide analog; TDF, tenofovir disoproxil fumarate; TAF, tenofovir alafenamide fumarate; TMF, tenofovir amibufenamide fumarate; EC_50_, 50% effective concentration; HBV, hepatitis B virus; TFV-DP, tenofovir diphosphate; PCA, principal component analysis; OPLS-DA, orthogonal partial least squares-discriminant analysis; SUS-plot, shared and unique structure-plot analysis; ESI, electrospray ionization; SGF, simulated gastric fluid; SIF, simulated intestinal fluid; P_*eff*_, permeability coefficient.
